# Identification and Validation of an Energy Metabolism-Related lncRNA-mRNA Signature for Lower-Grade Glioma

**DOI:** 10.1155/2020/3708231

**Published:** 2020-07-27

**Authors:** Jingwei Zhao, Le Wang, Bo Wei

**Affiliations:** ^1^Departments of Neurosurgery, The Third Hospital of Jilin University, Changchun, Jilin 130033, China; ^2^Ophthalmology, The First Hospital of Jilin University, Jilin University, Changchun, Jilin 130021, China

## Abstract

Energy metabolic processes play important roles for tumor malignancy, indicating that related protein-coding genes and regulatory upstream genes (such as long noncoding RNAs (lncRNAs)) may represent potential biomarkers for prognostic prediction. This study will develop a new energy metabolism-related lncRNA-mRNA prognostic signature for lower-grade glioma (LGG) patients. A GSE4290 dataset obtained from Gene Expression Omnibus was used for screening the differentially expressed genes (DEGs) and lncRNAs (DELs). The Cancer Genome Atlas (TCGA) dataset was used as the prognosis training set, while the Chinese Glioma Genome Atlas (CGGA) was for the validation set. Energy metabolism-related genes were collected from the Molecular Signatures Database (MsigDB), and a coexpression network was established between energy metabolism-related DEGs and DELs to identify energy metabolism-related DELs. Least absolute shrinkage and selection operator (LASSO) analysis was performed to filter the prognostic signature which underwent survival analysis and nomogram construction. A total of 1613 DEGs and 37 DELs were identified between LGG and normal brain tissues. One hundred and ten DEGs were overlapped with energy metabolism-related genes. Twenty-seven DELs could coexpress with 67 metabolism-related DEGs. LASSO regression analysis showed that 9 genes in the coexpression network were the optimal signature and used to construct the risk score. Kaplan-Meier curve analysis showed that patients with a high risk score had significantly worse OS than those with a low risk score (TCGA: HR = 3.192, 95%CI = 2.182‐4.670; CGGA: HR = 1.922, 95%CI = 1.431‐2.583). The predictive accuracy of the risk score was also high according to the AUC of the ROC curve (TCGA: 0.827; CGGA: 0.806). Multivariate Cox regression analyses revealed age, IDH1 mutation, and risk score as independent prognostic factors, and thus, a prognostic nomogram was established based on these three variables. The excellent prognostic performance of the nomogram was confirmed by calibration and discrimination analyses. In conclusion, our findings provided a new biomarker for the stratification of LGG patients with poor prognosis.

## 1. Introduction

Lower-grade gliomas (LGG) that include World Health Organization (WHO) grade II and III diffuse gliomas are common infiltrative brain tumors in adults [1]. Although advances have been made for the treatment of LGG, including neurosurgical resection, chemotherapy, and radiotherapy, a considerable proportion of patients still experience recurrence and malignant transformation to high-grade glioblastoma multiforme (GBM; WHO grade IV) [2], leading to declines in their health-related quality of life [3] and eventual death [2]. This heterogeneity in the prognosis of patients with LGG highlights the necessity to develop effective biomarkers to early stratify the patients at high risk for poor outcomes and give preventative therapy.

In order to maintain the malignant characteristics (rapid proliferation, migration, and invasion), tumor cells (including gliomas) need to produce a large amount of energy [4]. It is well known that carbohydrate, lipid, and amino acid metabolic processes are the main sources for the production of adenosine triphosphate (ATP) [5]. Therefore, the expression changes in genes involved in these metabolic processes may be important molecular mechanisms for the progression of gliomas, and the genes may represent potential biomarkers for prognostic prediction. This theory has been demonstrated by some scholars. For example, Qi et al. extracted the fatty acid catabolic metabolism-related genes from Molecular Signatures Database (MsigDB) and then identified an 8-gene risk signature using the Least Absolute Shrinkage and Selection Operator (LASSO) regression analysis based on RNA-seq data from the Chinese Glioma Genome Atlas (CGGA) dataset and The Cancer Genome Atlas (TCGA) dataset. This risk signature was found to be an independent prognostic factor for patients with all grade gliomas (CGGA: hazard ratios (HR) = 4.0044, 95%confidence intervals (CI) = 2.7634‐5.8028; TCGA: HR = 1.7382, 95%CI = 1.0577‐2.8567) [6]. Wu et al. used the Cox proportional hazards model to screen a prognostic signature from the differential genes of lipid metabolism between LGG and GBM. Consequently, a nine-gene signature was obtained as a classifier, which was demonstrated to significantly distinguish the overall survival (OS) between the high- and low-risk group of CGGA and TCGA cohorts [7]. Univariate Cox regression analysis performed by Zhao et al. generated a signature including 45 glucose-related genes. This risk score was associated with the OS of patients in the CGGA (HR = 2.293, 95%CI = 1.471‐3.576) training dataset and TCGA (HR = 1.227, 95%CI = 1.000‐1.504) and GSE16011 (HR = 1.440; 95%CI = 1.016‐2.039) validation datasets [8]. Based on glioma datasets from TCGA, REMBRANDT (Repository for Molecular Brain Neoplasia Data), and GSE16011, Chen et al. established a glycolytic gene expression signature score by incorporating ten glycolytic genes. A high risk score was reported to predict poor prognosis for patients with GBM [9]. Zhou et al. obtained 587 energy metabolism-related genes (including 41 carbohydrate, 73 lipid, and 144 protein metabolisms) from MsigDB and overlapped with the 463 differentially expressed genes between LGG and GBM to develop a risk score. As a result, a 29-gene signature was identified, and its predictive accuracy can reach 87.2% [10]. However, energy metabolism-related prognostic biomarkers for LGG remain rarely reported.

In addition, long noncoding RNAs (lncRNAs), a class of noncoding transcripts > 200 nucleotides in length, had also been demonstrated to affect cancer progression by regulation of the energy metabolism genes and then the related processes [11]. For example, Cheng et al. identified that highly expressed lncRNA X-inactive specific transcript (XIST) may promote cell viability, migration, invasion, and resistance to apoptosis by increasing glucose uptake, with the mechanism referring to upregulation of glucose transporters GLUT1 and GLUT3 [12]. The study of He et al. revealed that upregulated lncRNA UCA1 may induce glycolysis and invasion in glioma cells by competitively binding to miR-182 and then influencing the downstream target (fructose-2,6-biphosphatase) of miR-182 [13]. These findings indicated that the metabolism-related lncRNAs may also have underlying prognostic values for LGG; nevertheless, no studies focused on the lncRNA signature until now. Thus, the goal of this study was to construct a new energy metabolism-related lncRNA-mRNA prognostic signature for LGG patients.

## 2. Materials and Methods

### 2.1. Dataset Mining

Three datasets obtained from Gene Expression Omnibus (GEO; http://www.ncbi.nlm.nih.gov/geo/), TCGA (https://gdc-portal.nci.nih.gov/; level 3), and CGGA (http://www.cgga.org.cn; level 3) were included in our study. GSE4290 in GEO was enrolled because it met the following inclusion criteria: (1) studying RNA expression profiles, (2) studying brain tissue samples, (3) the number of samples more than 50, and (4) consisting of LGG (*n* = 76) and normal controls (*n* = 23). GSE4290 was used for screening the differentially expressed RNAs (DERs). Samples of TCGA (*n* = 520) and CGGA (*n* = 431) were eligible if they (1) belonged to the LGG type and (2) provided the clinical survival outcomes because TCGA was used as the training set for constructing the prognosis model, while CGGA was used as the validation set to confirm the prognosis value of the established model.

### 2.2. Identification of Energy Metabolism-Related Genes

The mRNAs and lncRNAs in the GSE4290 dataset were reannotated by the HUGO Gene Nomenclature Committee (HGNC; http://www.genenames.org/) that includes the standard nomenclature for 4516 lncRNAs and 19,200 protein-coding genes [14].

The Linear Models for Microarray Data (LIMMA) method (version 3.34.7; https://bioconductor.org/packages/release/bioc/html/limma.html) [15] in the Bioconductor R package (version 3.4.1; http://www.R-project.org/) was used to identify differentially expressed genes (DEGs) and lncRNAs (DELs) between LGG and normal controls. The false discovery rate (FDR) < 0.05 and ∣logFC(fold change) | >1 served as the screening threshold. Clustering analysis was conducted by the Pheatmap package (version: 1.0.8; https://cran.r-project.org/web/packages/pheatmap) in R language.

Energy metabolism-related gene sets (REACTOME_METABOLISM_OF_AMINO_ACIDS_AND_DERIVATIVES, REACTOME_METABOLISM_OF_CARBOHYDRATES, and REACTOME_METABOLISM_OF_LIPIDS) were collected from MSigDB (version 7.0; http://software.broadinstitute.org/gsea/msigdb/). These genes were overlapped with the DEGs to obtain energy metabolism-related DEGs.

### 2.3. Identification of Energy Metabolism-Related lncRNAs

Energy metabolism-related lncRNAs were determined by constructing a coexpression network between DELs and energy metabolism-related DEGs. The coexpression pairs were selected by calculation of Pearson correlation coefficients (PCC) between lncRNAs and DEGs by cor.test function (https://stat.ethz.ch/R-manual/R-devel/library/stats/html/cor.test.html) in R. PCC > 0.6 revealed that a significant correlation existed. The network was visualized in the Cytoscape software (version 3.6.1; http://www.cytoscape.org/).

### 2.4. Function Enrichment Analysis

To illustrate the specific metabolic processes involved of the lncRNAs in the network, function enrichment analysis was performed for the genes coexpressed with lncRNAs using the Database for Annotation, Visualization and Integrated Discovery (DAVID) (version 6.8; http://david.abcc.ncifcrf.gov) [16] and Enrichr (https://amp.pharm.mssm.edu/Enrichr/) [17]. Significant Gene Ontology (GO) biological process terms and Kyoto Encyclopedia of Genes and Genomes (KEGG) pathways with *p* value < 0.05 were collected and visualized using the ggplot2 package (version 3.3.0; https://cran.r-project.org/web/packages/ggplot2) in R.

### 2.5. Signature Development and Validation

Based on the TCGA data, univariate and multivariate Cox regression analyses were sequentially performed to evaluate the prognostic ability of energy metabolism-related DELs and DEGs in the coexpression network using “survival” package in R (version, 2.41-1; http://bioconductor.org/packages/survivalr/), with *p* < 0.05 tested by log-rank testing as the statistical threshold. A Cox proportional hazards model based on the L1-penalized regularization regression algorithm in the penalized package (version, 0.9-5; http://bioconductor.org/packages/penalized/) [18, 19] was subsequently conducted in DELs and DEGs still significant after multivariate analysis to further screen the optimal signature combination. The expression differences of signature genes between LGG and GBM were subsequently identified in GSE4290 (71 vs. 81), CGGA (443 vs. 249), and TCGA (524 vs. 155) datasets via an unpaired *t*-test to verify their specificity, with *p* < 0.05 set as a statistical difference. Finally, the risk score was established as follows:

Prognostic risk score = ∑*β*_DERs_ × Exp_DERs_, where Exp_DERs_ is the expression levels of prognostic DERs and ∑*β*_DERs_ is the prognostic coefficients of DERs after LASSO analysis.

The LGG patients were divided into the high-risk group and low-risk group using the median risk score as the cut-off. The Kaplan-Meier curve and receiver operating characteristic (ROC) curve were used to assess the predictive ability of the energy metabolism-related signature. These analyses were performed for the training dataset (TCGA) and validation dataset (CGGA), respectively.

To validate if the risk score could be independent of other clinicopathological parameters, univariate and multivariate Cox regression analyses were performed using the training dataset, followed by the stratification analysis for clinical variables with *p* < 0.05 in multivariate analysis. Furthermore, a nomogram using the result of multivariate Cox regression analysis was constructed to predict the 3-year and 5-year OS. The performance of the nomogram was assessed by discrimination and calibration. The discrimination was determined by the area under the curve (AUC) of the ROC curve and concordance index (C-index). The calibration was evaluated by calibration curves, which shows the agreement between the predicted and observed survival probabilities.

## 3. Results

### 3.1. Identification of Energy Metabolism-Related DERs

**Figure 1:**
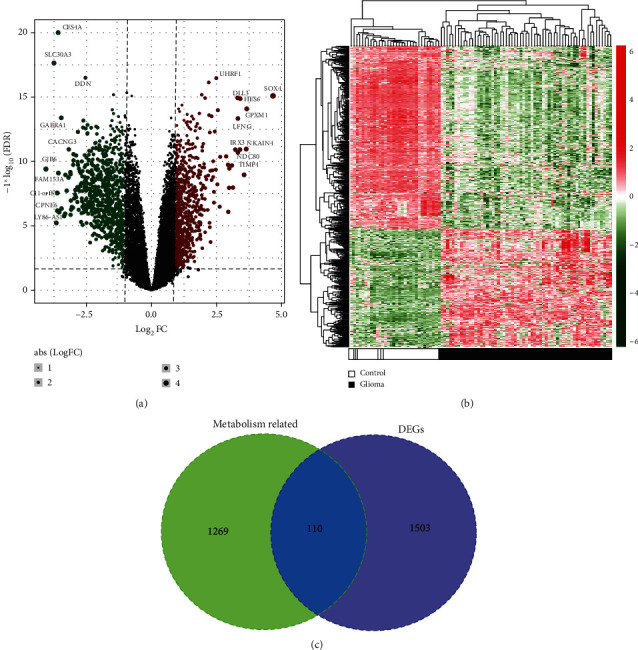
Identification of energy metabolism-related differentially expressed RNAs between lower-grade gliomas and normal brain tissues in the GSE4290 dataset. (a) Volcano plot to display the distribution of differentially expressed RNAs, which was performed by ggplot2. Green dots were downregulated RNAs; red dots were upregulated RNAs; black dots were RNAs not differentially expressed. FC: fold change; FDR: false discovery rate. (b) Heat map of differentially expressed RNAs, which was created by Pheatmap. Red indicated high expression; green indicated low expression. (c) Venn diagram to display the overlap between differentially expressed protein-coding genes (DEGs) and energy metabolism-related genes obtained from Molecular Signatures Database.

A total of 15,183 protein-encoding mRNAs and 576 lncRNAs were annotated in three included datasets. Based on the LIMMA method, 1613 mRNAs and 37 lncRNAs were found to be differentially expressed between LGG and normal brain tissues in the GSE4290 dataset (Figure 1(a)). Hierarchical clustering analysis showed that LGG samples could be clearly distinguished from normal samples according to the expressions of these DERs (Figure 1(b)).

By searching the MsigDB database, a total of 1403 energy metabolism-related genes were downloaded, including 293 for carbohydrate, 738 for lipid, and 372 for amino acids and derivative metabolism. These energy metabolism-related genes were then overlapped with the above 1613 DEGs, with 110 shown to be shared (Figure 1(c)), indicating that they were energy metabolism-related DEGs.

**Figure 2:**
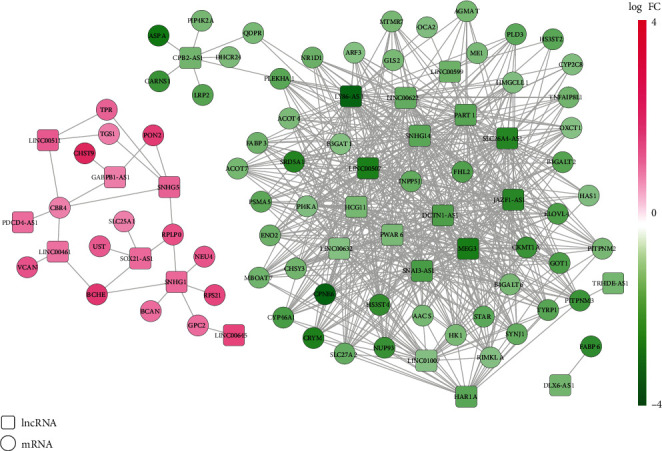
Identification of energy metabolism-related differentially expressed lncRNAs based on their coexpression with differentially expressed protein-coding mRNAs. Upregulated lncRNAs (square) and mRNAs (circle) were in red; downregulated lncRNAs (square) and mRNAs (circle) were in green. The coexpression pairs between lncRNAs and mRNAs were selected by calculation of Pearson correlation coefficients (PCC) by cor.test function. Only coexpression pairs with PCC > 0.6 were visualized.

**Figure 3:**
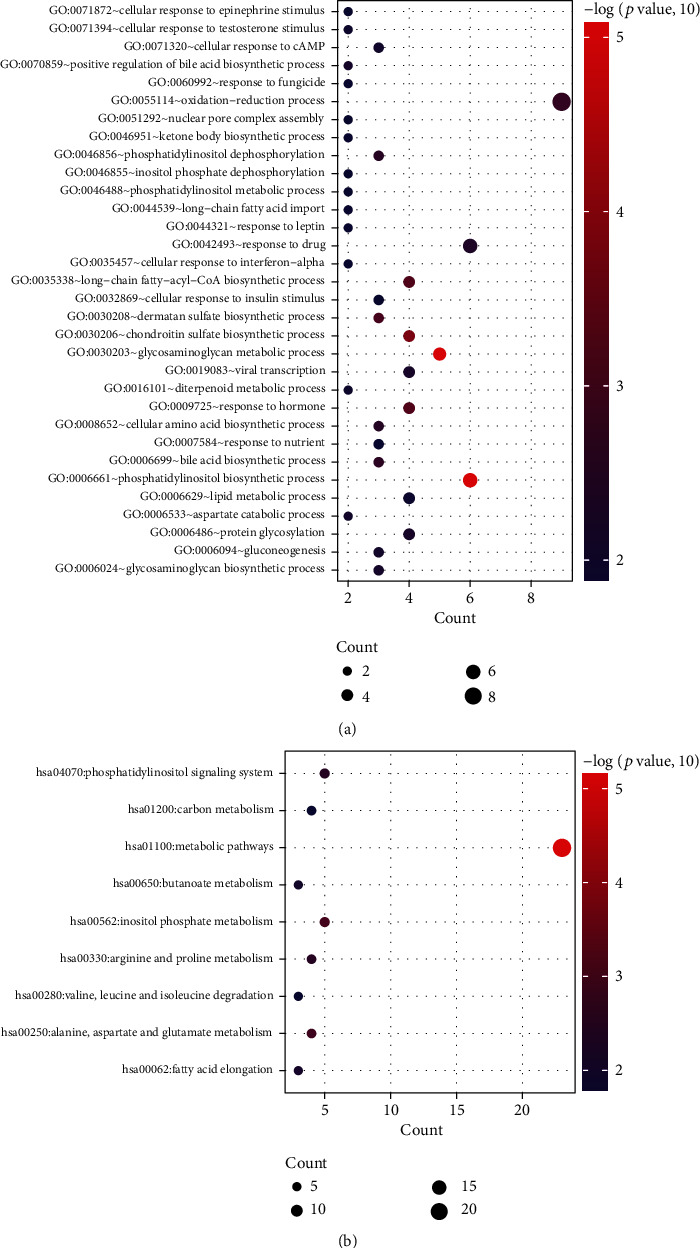
Function enrichment analysis for genes in the coexpression network by the Database for Annotation, Visualization and Integrated Discovery database. (a) Gene Ontology (GO) biological process terms; (b) Kyoto Encyclopedia of Genes and Genomes (KEGG). These plots were generated using the ggplot2 package.

By calculation of PCC, a total of 585 coexpression pairs between 27 DELs and 67 energy metabolism-related DEGs (such as GABPB1-AS1-PON2 (paraoxonase 2), HAR1A-CYP46A1 (cytochrome P450 family 46 subfamily A member 1)/HK1 (hexokinase 1), LINC00599-INPP5J (inositol polyphosphate-5-phosphatase J), SNAI3-AS1-INPP5J/HK1, and SNHG1-GPC2 (glypican 2)) (Figure 2), were obtained, indicating that these 27 DELs may be associated with the regulation of energy metabolism. Function enrichment analysis using DAVID (Figure 3; Table 1) and Enrichr (Supplementary Table 1) also showed that these 67 DEGs were enriched into energy metabolism-related GO biological process terms (such as GO:0030203~glycosaminoglycan metabolic process (GPC2), GO:0030208~dermatan sulfate biosynthetic process (UST, uronyl 2-sulfotransferase), GO:0055114~oxidation-reduction process (CYP46A1), GO:0046856~phosphatidylinositol dephosphorylation (INPP5J), GO:0006094~gluconeogenesis (SLC25A1, solute carrier family 25 member 1), GO:0006629~lipid metabolic process (FABP6, fatty acid binding protein 6), GO:0046488 phosphatidylinositol metabolic process (MBOAT7, membrane bound O-acyltransferase domain containing 7), and GO:0006631 fatty acid metabolic process (PON2)) and KEGG pathways (such as hsa01100: metabolic pathways (INPP5J, HK1), hsa01200: carbon metabolism (HK1), and glycerophospholipid metabolism (MBOAT7)). Thus, these 27 DELs and 67 DEGs were used for the following analyses.

### 3.2. Development of Energy Metabolism-Related DER-Based Risk Score

Univariate Cox regression analysis revealed that 13 out of 27 energy metabolism-related DELs and 27 out of 67 energy metabolism-related DEGs were significantly correlated with OS of LGG patients in the TCGA dataset. Then, they were entered into multivariate Cox regression. Five energy metabolism-related DELs and four energy metabolism-related DEGs were filtered as significant, independent prognostic factors. These 9 genes were further suggested to be the powerful prognostic indicators after LASSO regression analysis (Table 2). Also, there were significant expression differences in these 9 genes between LGG and GBM, indicating that they were specific signatures for LGG (Table 2). A risk score was constructed by combining the expression levels of these 9 genes with their LASSO coefficients as the following formula: (−0.05468 × expression of GABPB1-AS1) + (−0.64387 × expression of HAR1A) + (0.00904 × expression of LINC00599) + (−1.81399 × expression of SNAI3-AS1) + (0.29846 × expression of SNHG1) + (0.55872 × expression of FABP6) + (0.77305 × expression of MBOAT7) + (−0.40797 × expression of SLC25A1) + (−0.55501 × expression of UST).

**Figure 4:**
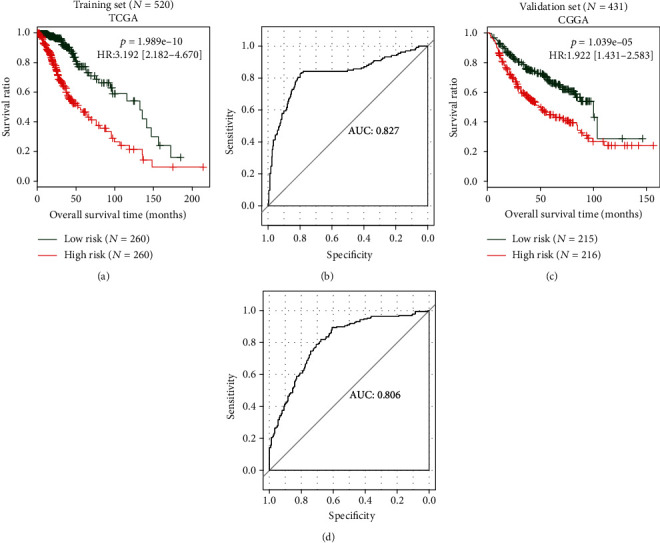
The prognostic performance assessment for the risk score model. (a, c) Kaplan-Meier survival curve analysis to show the overall survival difference between the high- and low-risk group of the training (a) and validation (c) datasets; (b, d) receiver operator characteristic (ROC) curves to demonstrate the predictive accuracy for the overall survival of patients in the training (b) and validation (d) datasets. CGGA: Chinese Glioma Genome Atlas; TCGA: The Cancer Genome Atlas; HR: hazard ratio; AUC: area under the ROC curve.

After calculation of the risk score for each patient in TCGA and CGGA datasets, the patients were dichotomized to the low-risk (<median) group and high-risk (≥median) group. Kaplan-Meier curve analysis showed that patients with a high risk score had significantly worse OS than those with a low risk score (TCGA: HR = 3.192, 95%CI = 2.182‐4.670, *p* = 1.989*e* − 10, Figure 4(a); CGGA: HR = 1.922, 95%CI = 1.431‐2.583, *p* = 1.039*e* − 05, Figure 4(c)). The predictive accuracy of the prognostic risk score was also demonstrated to be high according to the AUC of ROC curve (TCGA: 0.827, Figure 4(b); CGGA: 0.806, Figure 4(d)).

**Figure 5:**
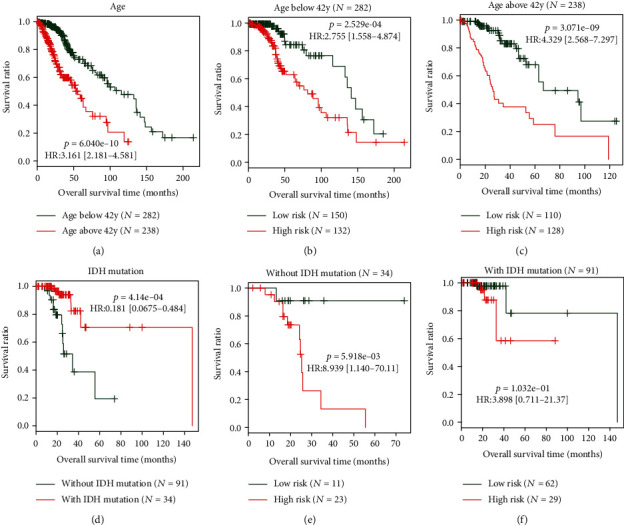
Stratification analysis based on age (a) and IDH1 mutation (d) which were also independent prognostic factors for overall survival in addition to the risk score. The Kaplan-Meier curve showed significant differences in overall survival between the high-risk group and the low-risk group in different ages (b, c) and IDH1 mutation status (e, f). HR: hazard ratio; y: year; IDH: isocitrate dehydrogenase.

**Figure 6:**
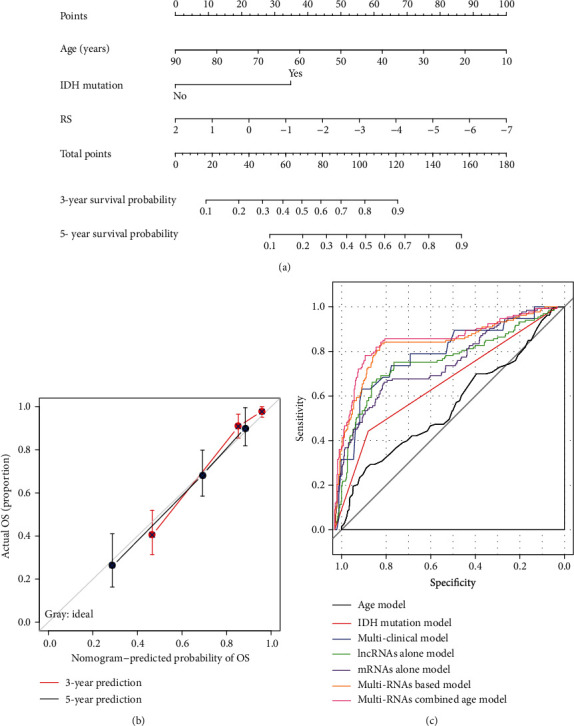
Establishment and assessment of a prognostic nomogram based on age, IDH1 mutation status, and the risk score. (a) A prognostic nomogram; (b) the calibration assessment by calibration curves; (c) the discrimination assessment by receiver operator characteristic curve. RS: risk score; IDH: isocitrate dehydrogenase.

To construct a prognostic nomogram, univariate and multivariate Cox regression analyses were performed for risk score and several clinical factors to explore all independent risk factors for OS. Univariate analysis demonstrated that age, histological type, isocitrate dehydrogenase 1 (IDH1) mutation, neoplasm histologic grade, radiation therapy, and risk score were significant prognostic factors. These significant variables further underwent multivariate analysis. As a result, age (Figure 5(a)), IDH1 mutation (Figure 5(d)), and risk score served as independent prognostic factors (Table 3). Also, stratification analysis showed that the risk score could further distinguish the prognosis of patients with the same age (<42 years, *p* = 2.529*e* − 04, Figure 5(b); ≥42 years, *p* = 3.071*e* − 09, Figure 5(c)) and IDH status (without mutation, *p* = 5.918*e* − 03, Figure 5(e); with mutation, *p* = 1.032*e* − 01, Figure 5(f)), implying that it is necessary to integrate the risk score into the clinical prognostic factors. Thus, a nomogram was then constructed based on these independent prognostic factors (Figure 6(a)). The excellent prognostic performance of the nomogram was confirmed by calibration (approximate to the 45-degree line for 3- and 5-year OS prediction) (Figure 6(b)) and discrimination (AUC = 0.845 and C‐index = 0.928; both higher than age, IDH status, and risk score model alone) (Table 4; Figure 6(c)).

## 4. Discussion

In this study, we, for the first time, attempted to develop an energy metabolism-related prognostic signature for LGG patients based on the lncRNAs and mRNAs. As a result, a prognostic risk score established by 9 energy metabolism-associated lncRNAs (GABPB1-AS1, HAR1A, LINC00599, SNAI3-AS1, and SNHG1)-mRNAs (FABP6, MBOAT7, SLC25A1, and UST) was generated. This risk score was demonstrated to be an independent prognostic factor for OS prediction, with the predictive accuracy reaching 82.7% for TCGA and 80.6% for the CGGA dataset, respectively, which seemed to be higher than the signature established by lncRNAs and mRNAs alone previously for LGG, such as Zhou et al. (29-mRNA, AUC = 79.1% for CGGA) [10], Ni et al. (25-mRNA, AUC = 77.1%) [20], Wang et al. (4-mRNA, AUC = 62.0%) [21], and Kiran et al. (8-lncRNA, AUC < 80%) [22]. This conclusion was also proved by our study (AUC = 0.827 vs. 0.747 for lncRNA alone model and 0.739 for mRNA alone model; C‐index = 0.812 vs. 0.702 for lncRNA alone model and 0.737 for mRNA alone model). Furthermore, the poor prognosis of LGG was traditionally determined according to clinical characteristics, including older age [23] and IDH1 nonmutational status [24, 25]. Thus, whether the risk score was superior or added additional prognostic value to these current clinical systems for prognosis prediction was also an important focus in the signature studies [10, 22, 26]. In the present study, we performed the stratification, AUC, C-index calculation, and nomogram analyses to confirm it. In line with previous studies [10, 22, 26], our results showed that the OS of patients with the same age and IDH1 mutation status can be further stratified by the risk score. Also, the AUC and C-index of risk score were obviously higher than age (AUC = 0.827 vs. 0.564; C‐index = 0.812 vs. 0.743) and IDH1 mutation status (AUC = 0.827 vs. 0.646; C‐index = 0.812 vs. 0.733). The prognostic performance was the highest (calibration plot: approximate to the 45-degree line for OS prediction; discrimination: AUC = 0.845; C‐index = 0.928) if age, IDH1 mutation, and the risk score were combined. These findings suggested that our identified combination (risk score+age+IDH1 mutation) may be a promising biomarker for clinical prediction of the outcomes in LGG patients.

Although our molecular prognostic signature was new for LGG patients, several lncRNAs included had been demonstrated to be related to the progression and prognosis for glioma. For example, the study of Luan et al. revealed that GABPB1-AS1 was a protective factor for the poor OS in patients with glioma (HR = 0.668, 95%CI = 0.494‐0.904) and GABPB1-AS1 may be involved in glioma by regulating autophagy-related genes [27]. Zou et al. reported that HAR1A was significantly downregulated in patients with GBM compared with nontumor controls (logFC = −2.873, *p* = 2.98*e* − 11). Multivariate analysis showed that low HAR1A expression was an independent prognosis factor for OS of glioma patients (HR = 1.6, *p* = 0.021) [28]. Fu et al. found that the expression of LINC00599 was reduced in LGG and GBM tissues as well as glioma cell lines compared with normal brain tissues or human astrocytes. Low LINC00599 expression was associated with poor disease-free survival and OS of glioma patients. *In vitro* study implied that overexpression of LINC00599 inhibited cell migration and invasion through blocking the epithelial-mesenchymal transition process [29]. Liu et al. observed that SNHG1 was overexpressed in glioma tissues and cell lines. *In vitro* and *in vivo* assays suggested that SNHG1 promoted glioma progression by functioning as a sponge for miR-194 and then inducing the high expression of pleckstrin homology like domain family A, member 1 (PHLDA1) [30]. Li et al. elucidated that SNHG1 may regulate the malignant behavior of glioma cells by binding to miR-154-5p or miR-376b-3p and then enhancing the expression of downstream target of both miR-154-5p and miR-376b-3p FOXP2 [31]. Kaplan-Meier analysis of Wang et al. showed that high expression of SNHG1 was significantly associated with poor OS. Functional studies demonstrated that knockdown of SNHG1 suppressed glioma cell proliferation and cell invasion and increased cell apoptosis [32]. In line with these studies, we also demonstrated that GABPB1-AS1 and SNHG1 were highly expressed, while HAR1A and LINC00599 were lowly expressed in LGG compared with normal brain tissues. They were all OS-related genes for LGG. However, their functions in glioma remain not well understood. In this study, we predicted that these four lncRNAs may play crucial roles in LGG by regulating the transcription of energy metabolism-related genes, including GABPB1-AS1-PON2, HAR1A-CYP46A1/HK1, LINC00599-INPP5J, and SNHG1-GPC2. The roles of these energy metabolism-related genes had been implicated for cancers. PON2 is a member of the paraoxonase family and had been demonstrated to exert an antioxidative function by improving mitochondrial efficiency to reduce reactive oxygen species production. Theoretically, PON2 should be downregulated in cancers exposed to anoxia. However, several studies showed that PON2 expression was obviously increased in gastric cancer [33] and bladder cancer [34] tissues compared with normal tissue samples. Overexpression of PON2 led to a significant increase in tumor cell proliferation and resistance to oxidative stress [34], while silencing of PON2 expression inhibited cancer cell proliferation, migration, and invasion [24]. Patients with high PON2 expression had shorter OS compared with those having low PON2 expression [23]. These findings indicated that high expression of PON2 may represent a protective stress response. It was reported that CYP46A1, a brain-specific enzyme that converts the cholesterol into 24(S)-hydroxycholesterol (24OHC), was significantly decreased in GBM samples compared with normal brain tissue. Low expression of CYP46A1 was associated with increasing tumor grade and poor prognosis in human gliomas [35]. Overexpression of CYP46A1 or the use of activator suppressed cell proliferation and *in vivo* tumor growth by increasing 24OHC levels [25]. Malignant tumors often rely on glycolysis to produce ATP (that is, Warburg effect), but not tricarboxylic acid cycle. Thus, glycolytic enzymes may play important roles for cancer. Hexokinase (HK) is the first rate-limiting enzyme to phosphorylate glucose to form glucose-6-phosphate (G-6-P). Theoretically, all hexokinase isozymes (HK1 to HK4) should be highly expressed for tumor initiation and maintenance. However, a study showed that HK2 was upregulated [36] but HK1 was downregulated to increase glycolysis and accelerate tumor growth and metastasis [37]. This result may be attributed to the underlying regulation between HK1 and HK2, which was also confirmed in the study of Tseng et al. (that is, knockdown of HK1 increased the HK2 level; silencing of HK2 elevated HK1 expression) [37]. INPP5J is a negative regulator for PI3K/AKT signaling and thus may function as a tumor suppressor, which was demonstrated in breast cancer [38], ovarian cancer [39], hepatocellular carcinoma [40], and oesophageal squamous cell carcinoma [41]. GPC2 is a member of the human glypican family of proteins that mediate neuronal cell adhesion and neurite outgrowth by attaching to the cell surface via a GPI anchor. Hereby, GPC2 protein is highly expressed in brain tumors, which had been validated in neuroblastoma [42, 43]. In accordance with these studies, we also identified that PON2 and GPC2 were upregulated, while CYP46A1, HK1, and INPP5J were downregulated in LGG compared with normal control (Supplementary Table 1).

The published study on SNAI3-AS1 was rarely reported, except one for hepatocellular carcinoma, in which highly expressed SNAI3-AS1 was shown to be correlated with shorter OS; knockdown of SNAI3-AS1 inhibited cell proliferation and metastasis, whereas inverse conclusions were obtained with overexpression of SNAI3-AS1 *in vitro*. Functional investigations showed that SNAI3-AS1 may affect tumorigenesis by inducing tumor epithelial to mesenchymal transition via regulating the UPF1/Smad7 signaling pathway [44]. Unfortunately, our results showed that SNAI3-AS1 was downregulated in LGG, suggesting that SNAI3-AS1 may be tissue-specifically expressed and a new target for LGG. Furthermore, we speculated that SNAI3-AS1 may function in LGG by coexpressing with HK1 and INPP5J as described above.

In addition to lncRNAs, four energy metabolism-related genes (FABP6, MBOAT7, SLC25A1, and UST) were also included into the prognostic signature, indicating their importance for LGG. Some genes had been demonstrated to be related to the development and progression of cancer previously. For example, a gastric cancer risk allele carrier was observed to have downregulated expressions of MBOAT7 [45]. Overexpression of mitochondrial citrate carrier (SLC25A1) was proved to be associated with reduced survival of lung cancer patients [46]. The mechanisms of SLC25A1 in cancer referred to its antioxidant defense and maintenance of the self-renewal capability of cancer stem cells [46, 47]. Knockdown of UST could significantly reduce migration and adhesion in mouse melanoma cells and pulmonary metastasis in mice [48]. However, no studies focused on glioma, suggesting they may also represent new biomarkers for LGG.

There were some limitations in this study. First, the expression of crucial lncRNAs and mRNAs should be verified with quantitative PCR, western blotting, or immunohistochemistry in LGG and normal brain tissues with larger sample size. Second, the prognosis ability of our risk score and combined model should be validated in newly hospitalized LGG patients. Third, *in vitro* and *in vivo* experiments are also essential to confirm the coexpression mechanisms of our identified lncRNAs (GABPB1-AS1-PON2, HAR1A-CYP46A1/HK1, LINC00599-INPP5J, and SNHG1-GPC2). Fourth, lncRNAs can function as competing endogenous RNAs (ceRNAs) to target mRNAs by sponging miRNAs. It may be a potential direction in the future to explore a signature established by lncRNA-miRNA-mRNA for prognosis prediction and reveal possible ceRNA mechanism axes for LGG as reported in other cancers [49]. Fifth, proteomics analysis also should be conducted in LGG and normal brain tissues in order to identify a protein alone or lncRNA-protein signature for prognosis prediction [50].

## 5. Conclusion

Our present study provided a new prognostic biomarker for LGG based on energy metabolism mechanisms. This prognostic signature consisted of five lncRNAs (GABPB1-AS1, HAR1A, LINC00599, SNAI3-AS1, and SNHG1) and four mRNAs (FABP6, MBOAT7, SLC25A1, and UST), which could classify patients into high- and low-risk subgroups exhibiting significantly different OS. Furthermore, this risk score was also combined with clinical characteristics (age, IDH1 mutation) to establish a prognostic nomogram, which may be more applicable for clinical use.

## Figures and Tables

**Table 1:** Function enrichment.

Category	Term	*p* value	Genes
Biology process	GO:0006661~phosphatidylinositol biosynthetic process	3.380*e* − 06	INPP5J, ARF3, SYNJ1, PI4KA, PIP4K2A, MTMR7
	GO:0030203~glycosaminoglycan metabolic process	5.100*e* − 06	B3GAT1, GPC2, CHST9, BCAN, VCAN
	GO:0030206~chondroitin sulfate biosynthetic process	1.310*e* − 04	CHSY3, CHST9, BCAN, VCAN
	GO:0009725~response to hormone	6.240*e* − 04	ME1, OXCT1, FHL2, DHCR24
	GO:0035338~long-chain fatty-acyl-CoA biosynthetic process	6.240*e* − 04	ACOT7, ELOVL4, SLC25A1, ACOT4
	GO:0030208~dermatan sulfate biosynthetic process	1.009*e* − 03	UST, BCAN, VCAN
	GO:0055114~oxidation-reduction process	2.379*e* − 03	ME1, TYRP1, CYP46A1, CYP2C8, QDPR, CBR4, SRD5A1, CRYM, DHCR24
	GO:0006699~bile acid biosynthetic process	3.136*e* − 03	CYP46A1, STAR, SLC27A2
	GO:0046856~phosphatidylinositol dephosphorylation	4.090*e* − 03	INPP5J, SYNJ1, MTMR7
	GO:0008652~cellular amino acid biosynthetic process	4.792*e* − 03	GLS2, ASPA, GOT1
	GO:0042493~response to drug	7.278*e* − 03	STAR, BCHE, OXCT1, FABP3, SRD5A1, AACS
	GO:0019083~viral transcription	1.015*e* − 02	RPLP0, NUP93, TPR, RPS21
	GO:0006486~protein glycosylation	1.040*e* − 02	B3GAT1, B3GALT2, B4GALT6, LRP2
	GO:0070859~positive regulation of bile acid biosynthetic process	1.192*e* − 02	NR1D1, STAR
	GO:0006024~glycosaminoglycan biosynthetic process	1.218*e* − 02	GPC2, HAS1, HS3ST2
	GO:0006094~gluconeogenesis	1.332*e* − 02	GOT1, ENO2, SLC25A1
	GO:0006533~aspartate catabolic process	1.587*e* − 02	ASPA, GOT1
	GO:0071320~cellular response to cAMP	1.829*e* − 02	STAR, RPLP0, SRD5A1
	GO:0016101~diterpenoid metabolic process	1.979*e* − 02	STAR, SRD5A1
	GO:0071394~cellular response to testosterone stimulus	1.979*e* − 02	SRD5A1, AACS
	GO:0006629~lipid metabolic process	2.485*e* − 02	CPNE6, AACS, LRP2, FABP6
	GO:0060992~response to fungicide	2.760*e* − 02	STAR, SRD5A1
	GO:0044539~long-chain fatty acid import	2.760*e* − 02	FABP3, SLC27A2
	GO:0044321~response to leptin	2.760*e* − 02	NR1D1, STAR
	GO:0046951~ketone body biosynthetic process	3.148*e* − 02	HMGCLL1, AACS
	GO:0007584~response to nutrient	3.523*e* − 02	STAR, OXCT1, AACS
	GO:0035457~cellular response to interferon-alpha	3.535*e* − 02	STAR, TPR
	GO:0032869~cellular response to insulin stimulus	3.788*e* − 02	GOT1, STAR, SRD5A1
	GO:0046855~inositol phosphate dephosphorylation	4.304*e* − 02	SYNJ1, MTMR7
	GO:0046488~phosphatidylinositol metabolic process	4.304*e* − 02	PITPNM3, SYNJ1
	GO:0051292~nuclear pore complex assembly	4.304*e* − 02	NUP93, TPR
	GO:0071872~cellular response to epinephrine stimulus	4.686*e* − 02	STAR, SRD5A1

KEGG pathway	hsa01100:metabolic pathways	9.150*e* − 06	ME1, PLD3, TYRP1, HMGCLL1, B3GALT2, CYP2C8, SYNJ1, PI4KA, QDPR, HK1, AGMAT, RIMKLA, ACOT4, GLS2, ASPA, CKMT1A, GOT1, CHSY3, INPP5J, ENO2, B4GALT6, MTMR7, DHCR24
	hsa00562:inositol phosphate metabolism	1.555*e* − 03	INPP5J, SYNJ1, PI4KA, PIP4K2A, MTMR7
	hsa00250:alanine, aspartate, and glutamate metabolism	1.894*e* − 03	GLS2, ASPA, GOT1, RIMKLA
	hsa04070:phosphatidylinositol signaling system	5.025*e* − 03	INPP5J, SYNJ1, PI4KA, PIP4K2A, MTMR7
	hsa00330:arginine and proline metabolism	5.263*e* − 03	CKMT1A, GOT1, AGMAT, CARNS1
	hsa00062:fatty acid elongation	1.343*e* − 02	ACOT7, ELOVL4, ACOT4
	hsa00650:butanoate metabolism	1.558*e* − 02	HMGCLL1, OXCT1, AACS
	hsa00280:valine, leucine, and isoleucine degradation	4.385*e* − 02	HMGCLL1, OXCT1, AACS
	hsa01200:carbon metabolism	4.608*e* − 02	ME1, GOT1, ENO2, HK1

**Table 2:** The optimal signature combination.

Symbol	Type	Expression (LGG vs. CK)		Expression (GBM vs. LGG)			Multivariate Cox regression analysis			LASSO coefficient
		logFC	FDR (GSE4290)	*p* value (GSE4290)	*p* value (CGGA)	*p* value (TCGA)	HR	95% CI	*p* value	
GABPB1-AS1	lncRNA	1.07	2.72*e* − 07	7.59*e* − 03	6.72*e* − 06	8.83*e* − 289	0.9946	0.9899-0.9994	2.846*e* − 02	-0.05468
HAR1A	lncRNA	-1.51	7.65*e* − 04	4.31*e* − 03	1.65*e* − 15	1.62*e* − 102	0.9938	0.988-0.9996	3.580*e* − 02	-0.64387
LINC00599	lncRNA	-1.3	4.80*e* − 05	3.64*e* − 02	2.36*e* − 06	2.62*e* − 105	1.0035	1.0001-1.007	4.628*e* − 02	0.00904
SNAI3-AS1	lncRNA	-1.76	9.51*e* − 08	7.96*e* − 03	3.12*e* − 10	1.71*e* − 258	0.9817	0.9701-0.9934	2.310*e* − 03	-1.81399
SNHG1	lncRNA	1.3	6.06*e* − 09	1.28*e* − 01	1.28*e* − 04	8.19*e* − 222	1.0054	1.0004-1.0104	3.328*e* − 02	0.29846
FABP6	mRNA	-2.18	1.13*e* − 07	2.94*e* − 01	4.34*e* − 02	1.42*e* − 128	1.0062	1.004-1.0085	6.270*e* − 08	0.55872
MBOAT7	mRNA	-1.27	3.92*e* − 09	8.01*e* − 01	1.11*e* − 03	0	1.0084	1.0017-1.0152	1.463*e* − 02	0.77305
SLC25A1	mRNA	1.03	2.39*e* − 09	4.05*e* − 03	5.96*e* − 12	0	0.9955	0.9911-0.9999	4.934*e* − 02	-0.40797
UST	mRNA	1.47	5.50*e* − 10	5.80*e* − 05	7.57*e* − 08	2.41*e* − 256	0.9951	0.9914-0.9989	1.177*e* − 02	-0.55501

LGG: lower-grade gliomas; GBM: glioblastoma multiforme; CK: normal control; CGGA: Chinese Glioma Genome Atlas; TCGA: The Cancer Genome Atlas; FC: fold change; FDR: false discovery rate; HR: hazard ratio; CI: confidence interval; LASSO: least absolute shrinkage and selection operator.

**Table 3:** Univariate and multivariate Cox regression analyses of clinical pathologic features for overall survival.

Variables	TCGA (*N* = 520)	Univariate analysis			Multivariate analysis		
		HR	95% CI	*p* value	HR	95% CI	*p* value
Age (years, mean ± SD)	42.84 ± 13.39	1.057	1.043-1.072	2.78**e** − 15	1.049	1.003-1.096	3.59**e** − 02
Gender (male/female)	286/234	1.145	0.811-1.617	4.40*e* − 01	—	—	—
Animal insect allergy history (yes/no/-)	16/289/215	0.831	0.202-3.428	7.92*e* − 01	—	—	—
Asthma history (yes/no/-)	22/346/152	0.894	0.409-1.956	7.76*e* − 01	—	—	—
Food allergy history (yes/no/-)	20/290/210	1.008	0.363-2.801	9.87*e* − 01	—	—	—
Hay fever history (yes/no/-)	38/304/178	0.488	0.177-1.347	1.24*e* − 01	—	—	—
Headache history (yes/no/-)	174/297/49	0.841	0.576-1.227	3.64*e* − 01	—	—	—
Histological type (astrocytoma/oligoastrocytoma/oligodendroglioma)	194/132/194	0.756	0.620-0.921	5.25**e** − 03	0.529	0.244-1.149	1.08*e* − 01
IDH1 mutation (yes/no/-)	91/34/395	0.181	0.0675-0.484	4.14**e** − 04	0.226	0.0729-0.701	9.98**e** − 03
Neoplasm histologic grade (G2/G3/-)	254/265/1	3.416	2.341-4.985	1.84**e** − 11	0.949	0.294-3.063	9.30*e* − 01
Radiation therapy (yes/no/-)	294/181/45	1.847	1.209-2.820	2.83**e** − 03	1.981	0.584-6.722	2.73*e* − 01
Targeted molecular therapy	268/200/52	1.389	0.955-2.019	8.08*e* − 02	—	—	—
Risk score (high/low)	260/260	3.192	2.182 -4.670	1.99**e** − 10	5.041	1.272-19.97	2.13**e** − 02
Death (dead/alive)	133/387	—	—	—	—	—	—
Overall survival days (months, mean ± SD)	32.97 ± 32.78	—	—	—	—	—	—

HR: hazard ratio; CI: confidence interval; SD: standard deviation; IDH: isocitrate dehydrogenase; TCGA: The Cancer Genome Atlas. Bold indicated the factors with statistical difference (*p* < 0.05).

**Table 4:** The performance of the nomogram assessed by discrimination parameters.

Model	AUC	C-index	*p* value	Specificity	Sensitivity
Age	0.564	0.743	0	0.889	0.278
IDH1 mutation	0.646	0.733	8.138*e* − 05	0.849	0.442
Multiclinical	0.779	0.8	1.278*e* − 06	0.877	0.632
lncRNA alone	0.747	0.702	9.97*e* − 14	0.832	0.662
mRNA alone	0.739	0.737	0	0.778	0.662
Multi-RNA based	0.827	0.812	0	0.798	0.827
Multi-RNA combined	0.845	0.928	0	0.863	0.782

AUC: area under the curve of receiver operating characteristic curve; C-index: concordance index; IDH: isocitrate dehydrogenase.

## Data Availability

All data were collected from GEO (GSE4290; http://www.ncbi.nlm.nih.gov/geo/), TCGA (https://gdc-portal.nci.nih.gov/), and CGGA (http://www.cgga.org.cn) databases.

## Supplementary Materials

Supplementary Materials Supplementary Table 1: function enrichment analysis for genes in the coexpression network by Enrichr.

## References

[1] Ostrom Q. T., Gittleman H., Farah P. (2013). CBTRUS Statistical Report: Primary Brain and Central Nervous System Tumors Diagnosed in the United States in 2006-2010. *Neuro-Oncology*.

[2] Chaichana K. L., McGirt M. J., Laterra J., Olivi A., Quiñones-Hinojosa A. (2010). Recurrence and malignant degeneration after resection of adult hemispheric low-grade gliomas. *Journal of Neurosurgery*.

[3] Okita Y., Narita Y., Miyahara R., Miyakita Y., Ohno M., Shibui S. (2015). Health-related quality of life in long-term survivors with grade II gliomas: the contribution of disease recurrence and Karnofsky Performance Status. *Japanese Journal of Clinical Oncology*.

[4] Basanta D., Simon M., Hatzikirou H., Deutsch A. (2008). Evolutionary game theory elucidates the role of glycolysis in glioma progression and invasion. *Cell Proliferation*.

[5] Libby C. J., Tran A. N., Scott S. E., Griguer C., Hjelmeland A. B. (2018). The pro-tumorigenic effects of metabolic alterations in glioblastoma including brain tumor initiating cells. *Biochimica et Biophysica Acta (BBA) - Reviews on Cancer*.

[6] Qi Y., Chen D., Lu Q., Yao Y., Ji C. (2019). Bioinformatic Profiling Identifies a Fatty Acid Metabolism-Related Gene Risk Signature for Malignancy, Prognosis, and Immune Phenotype of Glioma. *Disease Markers*.

[7] Wu F., Zhao Z., Chai R.‐. C. (2019). Prognostic power of a lipid metabolism gene panel for diffuse gliomas. *Journal of Cellular and Molecular Medicine*.

[8] Zhao S., Cai J., Li J. (2017). Bioinformatic profiling identifies a glucose-related risk signature for the malignancy of glioma and the survival of patients. *Molecular Neurobiology*.

[9] Chen C., Shi Y., Li Y. (2017). A glycolysis-based ten-gene signature correlates with the clinical outcome, molecular subtype and *IDH1* mutation in glioblastoma. *Journal of Genetics and Genomics*.

[10] Zhou Z., Huang R., Chai R. (2018). Identification of an energy metabolism-related signature associated with clinical prognosis in diffuse glioma. *Aging*.

[11] Tang J., Yan T., Bao Y. (2019). lncRNA GLCC1 promotes colorectal carcinogenesis and glucose metabolism by stabilizing c-Myc. *Nature Communications*.

[12] Cheng Z., Luo C., Guo Z. (2020). lncRNA-XIST/microRNA-126 sponge mediates cell proliferation and glucose metabolism through the IRS1/PI3K/Akt pathway in glioma. *Journal of Cellular Biochemistry*.

[13] He Z., You C., Zhao D. (2018). Long non-coding RNA UCA1/miR-182/PFKFB2 axis modulates glioblastoma-associated stromal cells-mediated glycolysis and invasion of glioma cells. *Biochemical and Biophysical Research Communications*.

[14] Povey S., Lovering R., Bruford E., Wright M., Lush M., Wain H. (2001). The HUGO gene nomenclature committee (HGNC). *Human Genetics*.

[15] Ritchie M. E., Phipson B., Wu D. (2015). *limma* powers differential expression analyses for RNA-sequencing and microarray studies. *Nucleic Acids Research*.

[16] Dennis G., Sherman B. T., Hosack D. A. (2003). DAVID: Database for Annotation, Visualization, and Integrated Discovery. *Genome Biology*.

[17] Kuleshov M. V., Jones M. R., Rouillard A. D. (2016). Enrichr: a comprehensive gene set enrichment analysis web server 2016 update. *Nucleic Acids Research*.

[18] Goeman J. J. (2010). *L*
_1_ penalized estimation in the Cox proportional hazards model. *Biometrical Journal*.

[19] Tibshirani R. (1997). The lasso method for variable selection in the Cox model. *Statistics in Medicine*.

[20] Ni J., Liu S., Qi F. (2020). Screening TCGA database for prognostic genes in lower grade glioma microenvironment. *Annals of Translational Medicine*.

[21] Wang W., Li J., Lin F., Guo J., Zhao J. (2020). Expression and prognostic value of mRNAs in lower grade glioma with MGMT promoter methylated. *Journal of Clinical Neuroscience*.

[22] Kiran M., Chatrath A., Tang X., Keenan D. M., Dutta A. (2019). A prognostic signature for lower grade gliomas based on expression of long non-coding RNAs. *Molecular Neurobiology*.

[23] Wang H., Wang X., Xu L., Zhang J., Cao H. (2020). Prognostic significance of age related genes in patients with lower grade glioma. *Journal of Cancer*.

[24] Auezova R., Ivanova N., Akshulakov S. (2019). Isocitrate dehydrogenase 1 mutation is associated with reduced levels of inflammation in glioma patients. *Cancer Management and Research*.

[25] Leu S., von Felten S., Frank S., Boulay J. L., Mariani L. (2016). *IDH* mutation is associated with higher risk of malignant transformation in low-grade glioma. *Journal of Neuro-Oncology*.

[26] Zhang C., Yu R., Li Z. (2019). Comprehensive analysis of genes based on chr1p/19q co-deletion reveals a robust 4-gene prognostic signature for lower grade glioma. *Cancer Management and Research*.

[27] Luan F., Chen W., Chen M. (2019). An autophagy-related long non-coding RNA signature for glioma. *FEBS Open Bio*.

[28] Zou H., Wu L. X., Yang Y., Li S., Zhou H. H. (2017). lncRNAs PVT1 and HAR1A are prognosis biomarkers and indicate therapy outcome for diffuse glioma patients. *Oncotarget*.

[29] Fu Q., Li S., Zhou Q., Yalikun K., Yisireyili D., Xia M. (2019). Low LINC00599 expression is a poor prognostic factor in glioma. *Bioscience Reports*.

[30] Liu L., Shi Y., Shi J. (2019). The long non-coding RNA SNHG1 promotes glioma progression by competitively binding to miR-194 to regulate PHLDA1 expression. *Cell Death & Disease*.

[31] Li H., Xue Y., Ma J. (2019). SNHG1 promotes malignant biological behaviors of glioma cells via microRNA-154-5p/miR-376b-3p- FOXP2- KDM5B participating positive feedback loop. *Journal of Experimental & Clinical Cancer Research*.

[32] Wang Q., Li Q., Zhou P. (2017). Upregulation of the long non-coding RNA SNHG1 predicts poor prognosis, promotes cell proliferation and invasion, and reduces apoptosis in glioma. *Biomedicine & Pharmacotherapy*.

[33] Wang X., Xu G., Zhang J. (2019). The clinical and prognostic significance of paraoxonase-2 in gastric cancer patients: immunohistochemical analysis. *Human Cell*.

[34] Bacchetti T., Sartini D., Pozzi V., Cacciamani T., Emanuelli M. (2017). Exploring the role of paraoxonase-2 in bladder cancer: analyses performed on tissue samples, urines and cell culturess. *Oncotarget*.

[35] Han M., Wang S., Yang N. (2020). Therapeutic implications of altered cholesterol homeostasis mediated by loss of CYP46A1 in human glioblastoma. *EMBO Molecular Medicine*.

[36] Patra K. C., Wang Q., Bhaskar P. T. (2013). Hexokinase 2 is required for tumor initiation and maintenance and its systemic deletion is therapeutic in mouse models of cancer. *Cancer Cell*.

[37] Tseng P. L., Chen C. W., Hu K. H., Cheng H. C., Chang W. T. (2018). The decrease of glycolytic enzyme hexokinase 1 accelerates tumor malignancy via deregulating energy metabolism but sensitizes cancer cells to 2-deoxyglucose inhibition. *Oncotarget*.

[38] Ooms L. M., Binge L. C., Davies E. M. (2015). The inositol polyphosphate 5-phosphatase PIPP regulates AKT1-dependent breast cancer growth and metastasis. *Cancer Cell*.

[39] Zhu T., Yuan J., Wang Y., Gong C., Xie Y., Li H. (2015). miR-661 contributed to cell proliferation of human ovarian cancer cells by repressing INPP5J expression. *Biomedicine & Pharmacotherapy*.

[40] Jiang J., Zhang Y., Guo Y. (2015). MicroRNA-3127 promotes cell proliferation and tumorigenicity in hepatocellular carcinoma by disrupting of PI3K/AKT negative regulation. *Oncotarget*.

[41] Lin C., Liu A., Zhu J. (2014). miR-508 sustains phosphoinositide signalling and promotes aggressive phenotype of oesophageal squamous cell carcinoma. *Nature Communications*.

[42] Li N., Fu H., Hewitt S. M., Dimitrov D. S., Ho M. (2017). Therapeutically targeting glypican-2 via single-domain antibody-based chimeric antigen receptors and immunotoxins in neuroblastoma. *Proceedings of the National Academy of Sciences of the United States of America*.

[43] Bosse K. R., Raman P., Zhu Z. (2017). Identification of GPC2 as an oncoprotein and candidate immunotherapeutic target in high-risk neuroblastoma. *Cancer Cell*.

[44] Li Y., Guo D., Ren M., Zhao Y., He S. (2019). Long non-coding RNA SNAI3-AS1 promotes the proliferation and metastasis of hepatocellular carcinoma by regulating the UPF1/Smad7 signalling pathway. *Journal of Cellular and Molecular Medicine*.

[45] SKM H., Hess T., Becker J. (2018). Evidence for *PTGER4*, *PSCA*, and *MBOAT7* as risk genes for gastric cancer on the genome and transcriptome level. *Cancer Medicine*.

[46] Hlouschek J., Hansel C., Jendrossek V., Matschke J. (2018). The mitochondrial citrate carrier (SLC25A1) sustains redox homeostasis and mitochondrial metabolism supporting radioresistance of cancer cells with tolerance to cycling severe hypoxia. *Frontiers in Oncology*.

[47] Fernandez H. R., Gadre S. M., Tan M., Graham G. T., Avantaggiati M. L. (2018). The mitochondrial citrate carrier, SLC25A1, drives stemness and therapy resistance in non-small cell lung cancer. *Cell Death and Differentiation*.

[48] Nikolovska K., Spillmann D., Haier J., Ladányi A., Stock C., Seidler D. G. (2017). Melanoma cell adhesion and migration is modulated by the uronyl 2-O sulfotransferase. *PLoS One*.

[49] Zhang Y., Ye Q., He J. (2020). Recurrence-associated multi-RNA signature to predict disease-free survival for ovarian cancer patients. *BioMed Research International*.

[50] Guo W., Sun S., Guo L. (2020). Elevated TOP2A and UBE2C expressions correlate with poor prognosis in patients with surgically resected lung adenocarcinoma: a study based on immunohistochemical analysis and bioinformatics. *Journal of Cancer Research and Clinical Oncology*.

